# Moonlighting transcriptional activation function of a fungal sulfur metabolism enzyme

**DOI:** 10.1038/srep25165

**Published:** 2016-04-28

**Authors:** Elisabetta Levati, Sara Sartini, Angelo Bolchi, Simone Ottonello, Barbara Montanini

**Affiliations:** 1 Biochemistry and Molecular Biology Unit, Laboratory of Functional Genomics and Protein Engineering, Department of Life Sciences, University of Parma, Parco Area delle Scienze 23/A, 43124 Parma, Italy

## Abstract

Moonlighting proteins, including metabolic enzymes acting as transcription factors (TF), are present in a variety of organisms but have not been described in higher fungi so far. In a previous genome-wide analysis of the TF repertoire of the plant-symbiotic fungus *Tuber melanosporum*, we identified various enzymes, including the sulfur-assimilation enzyme phosphoadenosine-phosphosulfate reductase (PAPS-red), as potential transcriptional activators. A functional analysis performed in the yeast *Saccharomyces cerevisiae*, now demonstrates that a specific variant of this enzyme, PAPS-red A, localizes to the nucleus and is capable of transcriptional activation. TF moonlighting, which is not present in the other enzyme variant (PAPS-red B) encoded by the *T. melanosporum* genome, relies on a transplantable C-terminal polypeptide containing an alternating hydrophobic/hydrophilic amino acid motif. A similar moonlighting activity was demonstrated for six additional proteins, suggesting that multitasking is a relatively frequent event. PAPS-red A is sulfur-state-responsive and highly expressed, especially in fruitbodies, and likely acts as a recruiter of transcription components involved in S-metabolism gene network activation. PAPS-red B, instead, is expressed at low levels and localizes to a highly methylated and silenced region of the genome, hinting at an evolutionary mechanism based on gene duplication, followed by epigenetic silencing of this non-moonlighting gene variant.

There is an increasing interest for multifunctional, especially “moonlighting” proteins, defined as proteins endowed with two or more, often unrelated functions associated to a single polypeptide chain^1^,^2^. Moonlighting proteins have been documented in a variety of organisms ranging from bacteria and yeast to humans^2^,^3^,^4^,^5^,^6^, but not in filamentous fungi so far. The reasons for the growing interest in moonlighting proteins have mainly to do with their inherent biological curiosity, the challenge they pose in genome/proteome annotation and their possible implications in biological circuitry analysis, *de novo* protein design and human diseases^7^,^8^. Multiple, apparently unrelated functions (e.g., cytosolic enzymes also acting as structural, chaperone/scaffold, cell motility-related or transport proteins) have been documented for moonlighting proteins^1^. In some cases, a strict taxonomic specificity of moonlighting activities has also been documented (see^1^ for a review). For example, different metabolic enzymes have been recruited as structural proteins of the eye lens (“crystallins”) in a strictly species-specific manner^9^.

A special case in point is represented by metabolic enzymes that moonlight as transcription factors, specifically designated as “trigger enzymes”^5^ or “metabolism-related transcription factors”^10^, i.e., proteins with the ability to couple metabolic state sensing with gene expression regulation, thus coordinating cell activity and adaptation in a metabolic signal-dependent manner. The latter include a variety of metabolic enzymes and cofactors (e.g., acetyl-CoA, S-adenosyl methionine and NAD^+^) directly or indirectly involved in gene expression regulation, with different documented or purported roles such as DNA/RNA binding, modulatory interaction with selected transcription machinery components, co-activator/repressor function and chromatin remodeling^11^,^12^,^13^ (reviewed in^10^).

Following up to the sequencing and annotation of the genome of the black truffle *Tuber melanosporum*, a filamentous mycorrhizal ascomycetous fungus, we carried out a genome-wide *in silico* and functional screening of the *Tuber* proteome searching for proteins endowed with transcription factor (TF) activity^14^. Given the as yet poor genetic tractability of truffles, this screening, named “transcription activator trap” (TAT)^15^,^16^, was conducted in the yeast *Saccharomyces cerevisiae*. It used as readout the ability of polypeptides representative of the entire *Tuber* proteome to confer reporter gene transactivation capacity to a deletion derivative of the yeast TF Gal4, capable of DNA-binding but lacking transactivation capacity. In this way, we functionally validated approximately one-fifth (37 out of 201) of the *in silico* predicted *T. melanosporum* TFs, but also identified 43 polypeptides whose potential ability to act as transcriptional activators had not been described before. The latter group comprised six metabolic enzymes, with a prevalence of dehydrogenases/reductases. These included PhosphoAdenosine-PhosphoSulfate reductase (PAPS-red), a key enzyme of the sulfur assimilation pathway, responsible for activated sulfate (PAPS) reduction and sulfite formation (see Fig. 1a).

At variance with other fungi, the *T. melanosporum* genome encodes two PAPS reductase enzymes, designated as PAPS-red A and B. The former enzyme (PAPS-red A), which was retrieved as a potential transcriptional moonlighter in our TAT screen^14^, is expressed at high levels in fruiting bodies, where sulfur assimilation is also involved in the production of secondary sulfur metabolites (S-Volatile Organic Compounds; S-VOCs) as components of the truffle aroma^17^.

Focusing on this enzyme, we show here that although devoid of a conventional (*in silico* predictable) nuclear localization signal (NLS), *T. melanosporum* PAPS-red A has an autonomous nuclear localization capacity. As revealed by functional comparison with PAPS-red enzymes from two unrelated ascomycetes (*S. cerevisiae* and *Neurospora crassa*) as well as with the *T. melanosporum* PAPS-red B enzyme, transcriptional moonlighting appears to be a unique property of PAPS-red A. Transcriptional activation capacity is associated to a transplantable, 23 amino acids C-terminal polypeptide extension, containing a peculiar alternation of hydrophilic and hydrophobic amino acids, as previously observed in several eukaryotic TFs, including yeast Gal4^18^,^19^.

The present work thus identifies a novel transcriptional moonlighting enzyme whose “second job” TF activity may be instrumental to the fine tuning of sulfur metabolism-related genes in an organism with a high reduced-sulfur demand such as *T. melanosporum*. We also document the potential transcriptional moonlighting activity of six additional black truffle proteins, three of which have an enzymatic metabolic activity as their “first job”.

## Results

### *T. melanosporum* PAPS-red A is a sulfur assimilation enzyme capable of transcriptional activation

**Figure 1:**
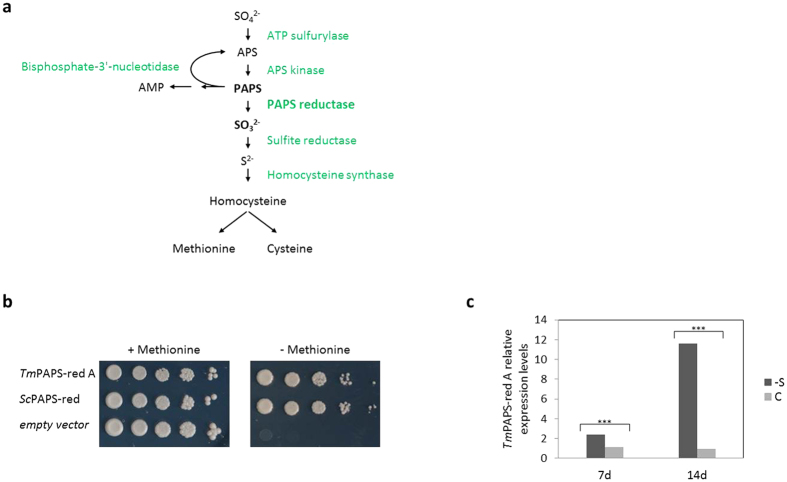
Functional and expression characterization of *T. melanosporum* PAPS reductase. (**a**) Schematic representation of the reductive sulfur assimilation pathway. Metabolic precursors/products and associated enzymes are shown in *black* and *green* respectively; PAPS reductase and its substrate and product are in *bold*. (**b**) Functional complementation of a *S. cerevisiae* PAPS-reductase *met16*Δ strain by the homologous Met16 enzyme (*ScPAPS-red*) and by *T. melanosporum* PAPS-red A (*TmPAPS-red A*). (**c**) PAPS red A expression levels in *T. melanosporum* mycelia grown on complete SSM (control) or sulfate-deprived synthetic medium for 7 or 14 days, relative to t_0_ mycelia determined by Real Time-RT-PCR; time-matched data-points derived from control, SSM-grown mycelia were used as reference; ***p-value < 0.01.

PAPS reductase catalyzes the second step of reductive sulfur assimilation and is responsible for the conversion of activated sulfate (PAPS) to sulfite (Fig. 1a). In the *T. melanosporum* genome it is encoded by two paralogous genes designated as PAPS-red A (GSTUMT00002663001) and PAPS-red B (GSTUMT00001561001), which are 99% and 98% identical at the nucleotide and amino acid sequence level, respectively. Both PAPS-red genes, whose nucleotide sequence identity (98.8%) extends to 3 kb upstream to the coding region, are composed of three exons with conserved exon-intron junctions. This extensive similarity suggests that both PAPS-red gene products are likely active catalytically. However, while PAPS-red B is located in a transposon-rich, highly methylated region of the *T. melanosporum* genome^20^ and is very poorly expressed, PAPS-red A is expressed at high levels, especially in fruiting bodies (see Supplementary Fig. S1, for a summary of RNAseq data).

In keeping with the lack of expression of PAPS-red B, four independent PAPS-red A clones, but no PAPS-red B sequence, were isolated from our previous TAT screen^14^. We thus focused on this enzyme form and cloned its full-length cDNA, corresponding to an N-terminally 35 aa extended version of the originally mispredicted GSTUMT00002663001 gene model. This cDNA was used to verify the catalytic competence of the predicted PAPS-red A enzyme by testing its ability to suppress the methionine auxotrophy phenotype of a *S. cerevisiae* mutant strain lacking the orthologous, PAPS-red encoding gene *MET16*. As shown in Fig. 1b, *T. melanosporum* PAPS-red A effectively complemented the biosynthetic defect of the *met16*Δ mutant strain and restored its ability to grow on synthetic medium lacking exogenously supplied methionine.

A common feature of PAPS reductase and other sulfate assimilation genes in various organisms is their upregulation in response to sulfate-starvation^21^,^22^,^23^,^24^. This also applies to PAPS-red A, whose mRNA levels increase more than 10-fold in sulfate-starved *T. melanosporum* mycelia (Fig. 1c). Additionally, PAPS red A expression levels are 14-fold more elevated in a high sulfur-demand tissue such as *Tuber* fruitbodies compared to free-living mycelia cultured under S-sufficient conditions (Supplementary Fig. S1). The above results indicate that *Tuber* PAPS-red A is not only catalytically competent and capable of functionally replacing the well-characterized yeast enzyme, but it is also tightly regulated in a life-cycle stage and sulfur state-dependent manner.

**Figure 2:**
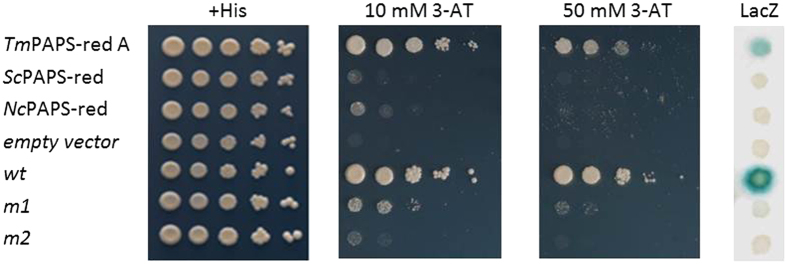
Comparative analysis of the transcriptional activation capacity of *T. melanosporum* PAPS-red A and of the orthologous PAPS reductase enzymes from yeast and *N. crassa*. β-galactosidase activity, histidine prototrophy and resistance to different concentrations of 3-AT were used to assay *LacZ* and *HIS3* reporter gene expression in yeast cells transformed with the corresponding Gal4 DBD-PAPS-red fusions as indicated; *TmPAPS-red A*: *T. melanosporum* PAPS reductase A, *ScPAPS-red*: *S. cerevisiae* PAPS reductase (Met16), *NcPAPS-red*: *N.crassa* PAPS reductase. All constructs were cloned in the pDEST32 vector; histidine-supplemented (+*His*) plates and empty pDEST32 vector transformants were used as general controls; *wt*, *m1* and *m2* are internal assay controls (see ‘Methods’ for details).

To verify the moonlighting transcriptional activity of PAPS-red A, we conducted an independent set of “transcription activator trap” assays using a newly assembled fusion construct containing the full-length PAPS-red A cDNA linked in-frame to the DNA-binding domain (DBD) of yeast Gal4. These new TAT assays confirmed our previous observations on the transcriptional activity of the *T. melanosporum* enzyme (Fig. 2). Specifically, the ability of PAPS-red A to act as an activation domain (AD) effectively trans-activating the expression of Gal4-dependent *LacZ* and *HIS3* reporter genes. In fact, as shown by the TAT assay results in Fig. 2, the Gal4-DBD-PAPS-red A fusion led to the production of well-detectable β-galactosidase enzyme levels and fully restored histidine prototrophy as well as resistance to high concentrations (up to 50 mM) of the His3 enzyme inhibitor 3-amino-triazole (3-AT).

In order to gain information on the general or organism-specific nature of the transcriptional moonlighting activity of PAPS reductase, two additional constructs expressing Gal4-DBD-PAPS-red fusions containing the orthologous enzymes from *S. cerevisiae* and *N. crassa* were assembled and tested with the TAT assay. As further shown in Fig. 2, no growth on histidine-lacking medium containing 10 mM 3-AT was observed with the *S. cerevisiae* PAPS-red fusion, while only a slight growth at the lowest cell dilution and in the presence of the lowest 3-AT concentration (10 mM), but no growth in the presence of a higher 3-AT concentration (50 mM), were observed with a fusion construct bearing PAPS-red from *N. crassa*. Transactivation capacity, i.e. the ability to functionally replace the activation domain of yeast Gal4 in the recruitment of downstream components of the RNA Pol II transcription apparatus, thus appears to be a most prominent, and perhaps unique feature of the *T. melanosporum* PAPS-red A enzyme, rather than a common property of ascomycete PAPS-reductases.

### Autonomous nuclear translocation capacity of *T. melanosporum* PAPS-red A

In the TAT assay, the Gal4-DBD fusion partner contains the nuclear localization signal (NLS) of the yeast Gal4 activator. Furthermore, no putative NLS could be recognized by various signal search programs in the *T. melanosporum* PAPS-red A polypeptide. This raised the possibility that the observed transactivation capacity of the truffle enzyme may reflect, and be due to, forced accumulation into the nuclear compartment in association with the Gal4-DBD, rather than a genuine and physiologically significant nuclear activity.

**Figure 3:**
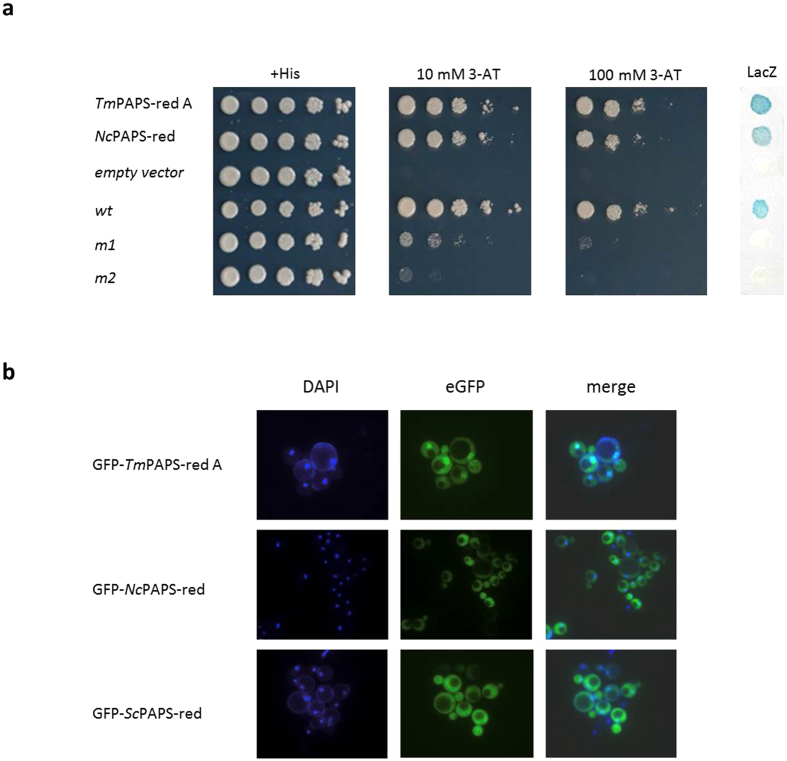
Autonomous nuclear localization capacity of *T. melanosporum* PAPS-red A. (**a**) NTT assay using growth on histidine-lacking plates supplemented with different concentrations of the 3-AT inhibitor (*HIS3*) and β-galactosidase activity (*LacZ*) as readouts. Both constructs (*TmPAPS-red A* and *NcPAPS-red*) were cloned in the pNIA-CEN-MBP vector and transformed into the L40 yeast strain; +*His*, *empty vector* and internal assay (*wt*, *m1* and *m2*) controls are the same as those described in Fig. 2 legend. (**b**) Subcellular localization of the indicated GFP-PAPS reductase fusion constructs; nuclear localization of the GFP-*Tm*-PAPS-red A fusion protein is apparent. DAPI staining was used to mark the nucleus.

We addressed this point with another yeast-based assay, named “nuclear transportation trap” (NTT)^25^,^26^, which measures the ability of a foreign (“query”) polypeptide to drive nuclear accumulation, and thus transcriptional activity, of an NLS-lacking fusion protein comprising the DBD of the bacterial regulator LexA fused to the activation domain of yeast Gal4. As shown in Fig. 3a, which compares the NTT assay performance of *Tuber* PAPS-red A with that of *Neurospora* PAPS reductase, both proteins appear to be capable of inducing basal levels of nuclear internalization, sufficient to support yeast growth in the absence of exogenously supplied histidine, under permissive conditions, i.e., at the minimum cell input dilution and in the presence of the lowest concentration (10 mM) of the 3-AT inhibitor. However, a considerably higher growth (up to the fourth cell input dilution) under more stringent (100 mM 3-AT) selection conditions and a stronger *LacZ* reporter gene signal were produced by the *Tuber* PAPS-red A- compared to the *Neurospora* PAPS-red-containing construct. This suggests that although both proteins contain cryptic nuclear localization signals capable of driving nuclear entry to different extents, considerably higher levels of nuclear internalization, and thus a more sustained reporter gene activation, are supported by *Tuber* PAPS-red A. As shown in Fig. 3b, this conclusion is also corroborated by the results of a fluorescence microscopy analysis indicating the dual, cytoplasmic and nuclear, localization of an N-terminal GFP fusion derivative of the *T. melanosporum* PAPS-red A enzyme, compared to the predominantly cytoplasmic localization of the corresponding *Neurospora* and *S. cerevisiae* GFP-PAPS-red fusion proteins.

### Transactivation capacity is associated to a transplantable C-terminal polypeptide extension of *T. melanosporum* PAPS-red A

**Figure 4:**
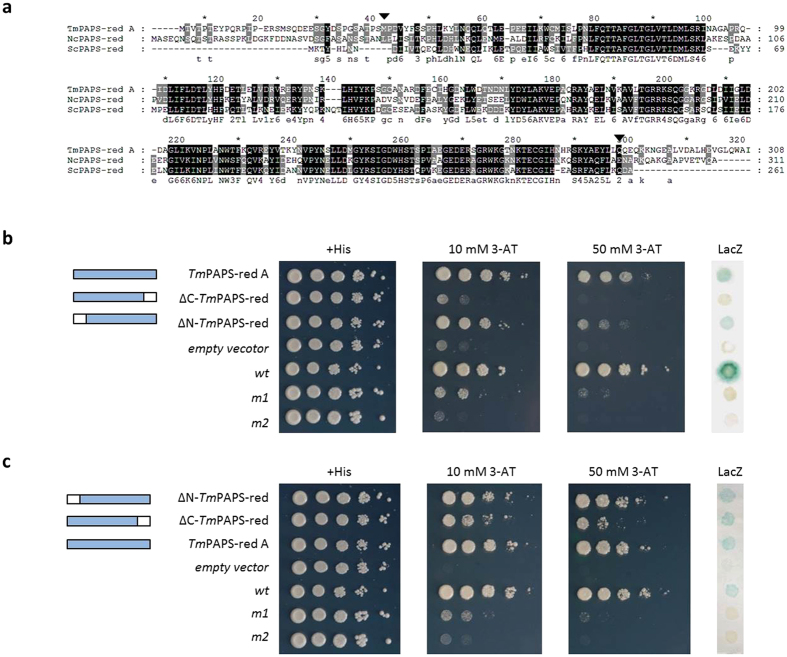
Functional dissection of *T. melanosporum* PAPS-red A. (**a**) Multiple sequence alignment of the *T. melanosporum*, *N. crassa* and *S. cerevisiae* PAPS-reductase predicted polypeptides; arrowheads indicate the boundaries of the N- and C-terminally deleted variants of *Tuber* PAPS-red A that were assayed for transcriptional activation and autonomous nuclear localization capacity. The indicated N- and C-terminal deletion derivatives of *Tuber* PAPS-red A were assayed for transcriptional activation (**b**) and autonomous nuclear localization capacity (**c**) using the same general (+*His* and *empty vector*) and assay-specific (*wt*, *m1* and *m2*) controls as described in Fig. 2 legend. *Tm*PAPS-red A: full-length *T. melanosporum* PAPS reductase A; ΔN-*Tm*PAPS-red: PAPS-red A deletant lacking the 35 N-terminal amino acids; ΔC-*Tm*PAPS-red: PAPS-red A deletant lacking the 23 C-terminal amino acids (see Figs 2 and 3 legends and “Methods” for further details).

Having demonstrated the apparently unique transcriptional moonlighting activity of *T. melanosporum* PAPS-red A, we next compared its polypeptide sequence with those of the homologous but transcriptionally inactive enzymes from yeast and *Neurospora*. As shown by the sequence alignment in Fig. 4a, the central portion of the enzyme, including the active site region, is highly conserved, with a minimum sequence similarity of 74%. Instead, fairly divergent N- and C-terminal extensions, ranging in size from 35 to 41 and 17 to 23 amino acids, respectively, are present in the *Tuber* and *Neurospora*, but not in the yeast PAPS reductase.

To gain insight on the possible functional significance of these sequence extensions, Gal4-DBD and LexA-Gal4-AD fusion derivatives of *Tuber* PAPS-red A lacking either the N-terminal or the C-terminal extension were assembled and tested by the TAT and the NTT assays. As shown in Fig. 4b, deletion of the N-terminal 35 amino acids extension caused a slight impairment of *LacZ* and *HIS3* transactivation capacity, only detectable in the presence of a high 3-AT concentration (*right panel*), whereas a complete loss of transactivation capacity was caused by removal of the C-terminal 23 amino acids extension. In contrast with the marked effect of C-terminal extension deletion on transactivation, only a slightly reduced ability to drive nuclear internalization was revealed by the NTT assay analysis of the C-terminus deletant compared to the N-terminal deletant and to the control construct bearing the full-length PAPS-red A sequence (Fig. 4c). The latter finding suggests that the cryptic NLS associated to PAPS-red A is located in the C-terminal part of the *T. melanosporum* enzyme core region.

**Figure 5:**
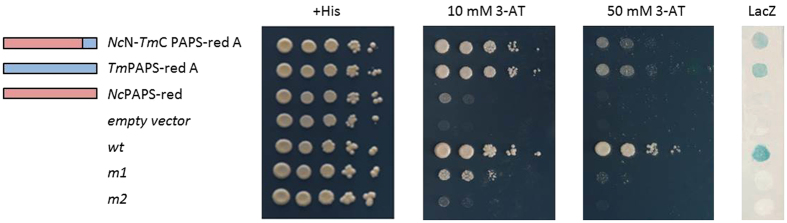
Addition of the C-terminal extension fragment of *T. melanosporum* PAPS-red A confers transactivation capacity to *Neurospora* PAPS reductase. TAT assay results obtained with the indicated Gal4-DBD-PAPS-red fusion constructs; *Nc*N-*Tm*C PAPS-red A: *Tuber* PAPS-red A C-terminal fragment (*blue bar*) fused to the core *Neurospora* PAPS-red enzyme (*red bar*). Full-length *T. melanosporum* PAPS-red A (*blue bar*) and *N. crassa* PAPS-red (*red bar*) constructs served as references and controls; the other controls (+*His*, *empty vector*, *wt*, *m1* and *m2*) are the same as described in Fig. 2 legend.

To further validate the transcriptional activation capacity of the *Tuber* PAPS-red A C-terminus, we directly fused the C-terminal 23 amino acids of PAPS-red A to the C-terminus of the Gal4DBD and tested the ability of the resulting chimeric protein to transactivate reporter gene expression. This minimal fusion construct turned out to be transactivation-incompetent, possibly due to the lack of structural constraining by the core region of the full-length PAPS-red A polypeptide. We thus tested the transactivation capacity of the same *Tuber* PAPS-red A C-terminal peptide fused to the core sequence of the transcriptionally inactive *Neurospora* enzyme (see Fig. 4a). As shown in Fig. 5, this chimeric protein construct (*Nc*N*-Tm*C PAPS-red A) bearing only the last 23 C-terminal amino acids of *Tuber* PAPS-red A, was indeed capable of activating *LacZ* and *HIS3* transcription, also conferring resistance to 50 mM 3-AT.

Altogether the above data -i.e., loss of PAPS-red A transactivation capacity upon C-terminal extension deletion and gain of transcriptional activation function by *Neurospora* PAPS reductase upon transplantation of the PAPS-red A C-terminal peptide- indicate the C-terminal 23 amino acids of *T. melanosporum* PAPS-red A as the key region required for the moonlighting transcription factor activity of this sulfur metabolic enzyme.

### Three hydrophilic to hydrophobic amino acid substitutions in the C-terminal peptide extension of PAPS-red A are required for transactivation function

**Figure 6:**
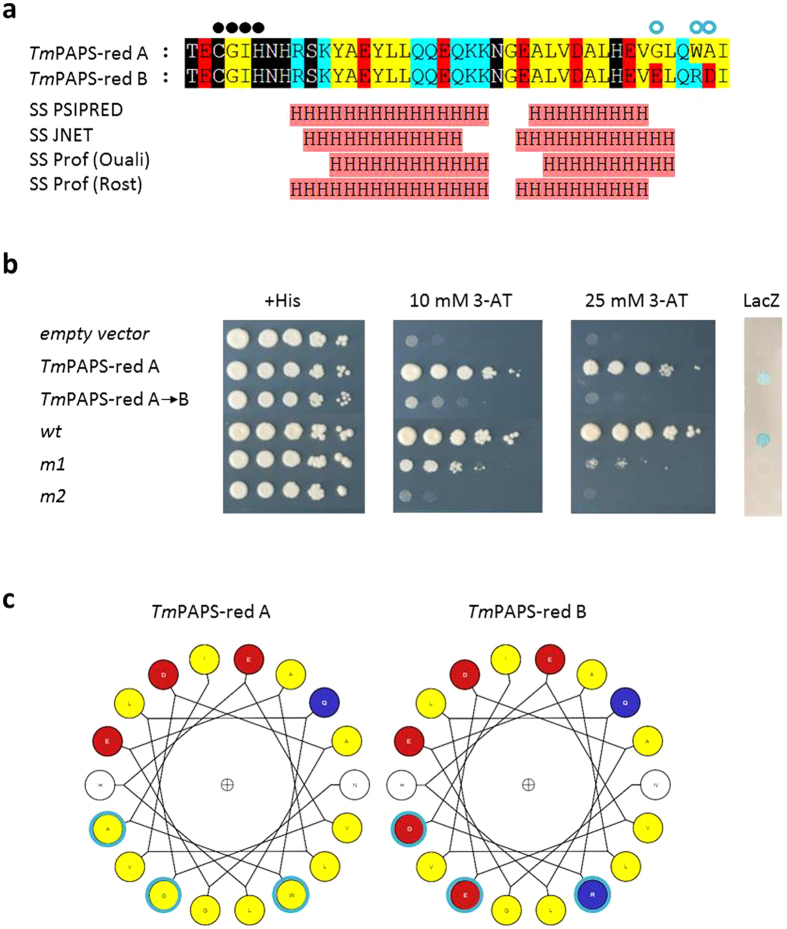
Three polar to hydrophobic C-terminus amino acid substitutions are sufficient to confer transcriptional activation capacity to the inactive *Tuber* PAPS-red B enzyme. (**a**) Alignment and predicted secondary structure (H: alpha-helices) of the C-terminal polypeptide extensions of PAPS-red A and B. Key active site residues and the three divergent amino acids that distinguish the two enzyme forms are indicated by filled *black* and empty blue circles, respectively. See ‘Methods’ for details. (**b**) TAT assay analysis of a PAPS-red A variant (*Tm*PAPS-red A->B), in which the three divergent hydrophobic amino acids of PAPS-red A were replaced by the three hydrophilic amino acids residues of PAPS-red B. Full-length wild-type PAPS-red A fused to the Gal4-DBD (*Tm*PAPS-red A) served as a reference and positive control for this experiment; the other controls (+*His*, *empty vector*, *wt*, *m1* and *m2*) were as described in Fig. 2 legend. (**c**) Helical wheel projections of the last 18 residues of PAPS-red A and B. Positively charged, negatively charged and non-polar amino acids are indicated in *blue*, *red*, and *yellow* respectively. The three amino acid residues that distinguish PAPS-red A from PAPS-red B are bordered in *light blue*.

PAPS-red B also comprises a 23 aa. C-terminal extension, which contains three of the five amino acid substitutions that distinguish this enzyme form from PAPS-red A, all corresponding to hydrophilic (PAPS-red B) to hydrophobic (PAPS-red A) amino acid replacements (Fig. 6a). We thus asked whether these amino acid changes might influence transactivation function. To address this question, we converted the three hydrophobic residues of PAPS-red A into the hydrophilic residues present at equivalent positions in PAPS-red B by site-directed mutagenesis. As revealed by the TAT assay data in Fig. 6b, substitution of these three amino acids caused a complete loss of transactivation capacity. This indicates that a specific amino acid sequence motif rather than simply the length of the C-terminal extension is critical for transactivation. This motif consists of an alternation of hydrophilic amino acid residues with stretches of hydrophobic amino acids and is compatible with an amphipathic α-helix structure (Fig. 6c). Similar motifs have previously been reported for the activation domains of several eukaryotic transcription factors^18^,^27^,^28^,^29^,^30^, where they have been shown to mediate interaction with different components of the RNA pol. II transcription machinery^19^,^31^.

## Discussion

By demonstrating the gene transactivation and autonomous nuclear localization capacity of black truffle PAPS-red A, this work adds one more member to the growing list of transcriptional function moonlighting enzymes and reveals a potentially novel metabolic context (i.e., reductive sulfur assimilation) for moonlighting activity. Initially considered as oddities and potential confounders of large-scale screening studies^32^, transcriptional moonlighting proteins are increasingly emerging as novel players in as yet poorly understood gene regulatory circuits, both as DNA binders^5^,^11^ and as secondary recruiters or modulators of specific transcription machinery components^33^,^34^,^35^ (reviewed by^4^,^10^).

Although never described in filamentous fungi so far, transcriptional activity is one of the most frequent “second jobs” of metabolic enzymes and it is thought to represent a potential new means of coordinating metabolic state with the modulation of specific gene networks^10^. Indeed, as outlined in Table 1, in a non-exhaustive TAT/NTT screening conducted in *T. melanosporum* we have also identified three enzymes plus three non-enzymatic proteins in addition to PAPS-red A as transcriptional moonlighters capable of autonomous nuclear entry.

Two main molecular mechanisms have been shown to underlie the moonlighting transcriptional activity of metabolic enzymes: DNA or RNA binding, leading to either site-specific recruitment of additional (downstream-acting) regulators or to transcript (de)stabilization^5^,^11^,^12^, and interaction with specific (free or DNA-preassembled) downstream-acting transcriptional components (e.g., co-activators or repressors) so to change their functional state and/or capacity to recognize their targets^4^,^10^.

Recombinantly expressed, GST-tagged *Tuber* PAPS-red A was found to be capable of interacting with DNA (non-specifically) in a gel-shift assay using a plasmid-derived DNA fragment as potential binding site (see Supplementary Fig. S2). However, further experiments probing the ability of GST-PAPS-red A to bind to double-stranded oligonucleotides displayed on a protein-binding array covering all possible combinations of 10 bp-long DNA binding sites^36^ failed to retrieve any specific hit. This apparent lack of DNA binding specificity is also supported by the absence in *Tuber* PAPS-red A of any recognizable DNA binding motif.

Even though we cannot entirely exclude the possibility that DNA binding may require a longer sequence or a multi-component network of protein-DNA contacts, we believe that the moonlighting transcriptional activity of PAPS-red A relies on protein-protein interaction with specific components (e.g., DNA-bound TFs or co-activators) of the sulfur metabolism transcriptional regulatory apparatus, rather than on direct DNA binding.

This target specificity is indirectly supported by the observed S-state responsiveness of PAPS-red A expression (Fig. 1c) and by the high abundance of PAPS-red A transcripts in a sulfur metabolism-proficient tissue (including secondary S-metabolism responsible for S-VOC production^17^) such as the truffle fruiting body, compared to free-living mycelium (Supplementary Fig. S1)^37^. In this regard, *T. melanosporum* PAPS-red A may resemble some sugar metabolism enzymes (e.g., hexokinase and galactokinase) that in *S. cerevisiae* and other yeasts, as well as in higher eukaryotes, have been shown to moonlight, often in a species-specific manner, as non-DNA-binding transcriptional regulators^4^,^34^,^38^,^39^.

In keeping with a gene transactivation function of PAPS-red A, is also the fact that as previously observed for many eukaryotic transcriptional activators^18^,27–29 such function critically depends on a C-terminally located polypeptide region displaying a peculiar alternation of hydrophilic and hydrophobic amino acids. Originally identified in the yeast transcriptional activator Gal4 as responsible for interaction with multiple transcription components (e.g., three different subunits of the general transcription factor TFIID, TFIIB, the mediator subunit Gal11, the HAT complexes SAGA and NuA4, plus the chromatin remodeler Swi/Snf^31^), this AD sequence feature has subsequently been documented in several eukaryotic transcription factors, where it has been shown to promote interaction with various downstream-acting transcriptional effectors, including chromatin remodeling and HAT-containing complexes^19^,^40^,^41^,^42^,^43^. Transplantability, as observed for the PAPS-red A C-terminal peptide, and lack of stringent structural requirements are additional features of these “simple” (and rather promiscuous) activation domains. Of note, the last 33 amino acids of PAPS-red A, which include its activating C-terminal extension, are predicted to form an α-helix (Fig. 6a,c) that is only two amino acids apart from the enzyme active site. Furthermore, based on the 3D structure of the orthologous yeast enzyme, this α-helix is predicted to be exposed on the surface of the protein.

A final question regards the taxonomic rank specificity of PAPS reductase moonlighting activity and its mode of evolution. We have shown that transcriptional activity is not present or extremely weak in the case of the PAPS-red from the ascomycetes *S. cerevisiae* and *N. crassa*, respectively, and that relatively small (easily evolvable) sequence changes can convey such an activity to otherwise transcriptionally inactive PAPS-red enzymes. It is presently not known whether transcriptional moonlighting is associated to PAPS reductase enzymes from closely related *Tuber* species. We note, however, that the PAPS-red C-terminus of the whitish truffle *T. borchii* (accession number AF462035) as well as those of two other truffle species (*T. aestivum* and *T. magnatum*; Pezizomycete Pan-genome consortium, personal communication) more closely resemble the transcriptionally inactive PAPS-red B rather than the PAPS-red A enzyme. Also worth of note is the presence of two PAPS-red genes in *T. melanosporum* but not in any of the other *Tuber* species sequenced so far (Pezizomycete Pan-genome consortium, personal communication). As in other cases of moonlighting protein variants^44^, acquisition of transcriptional moonlighting activity by *T. melanosporum* PAPS-red A has likely involved a duplication of the gene coding for the non-moonlighting PAPS-red B. What is quite unusual, however, and perhaps more typical of complex eukaryotic genomes, is the fact that the PAPS-red B gene was transcriptionally inactivated via incorporation into a transposon-rich, highly methylated and silenced region of the *T. melanosporum* genome^20^. While transcriptional silencing of the non-moonlighting PAPS-red B gene might be aimed at avoiding cellular competition with the transcriptionally active PAPS-red A enzyme, the specific metabolic advantages conferred by a transcriptional moonlighting PAPS reductase enzyme in a particular truffle species such as *T. melanosporum* remain to be determined.

## Methods

### Functional complementation assay

To test the catalytic competence of *T. melanosporum* PAPS reductase, full-length PAPS-red A was cloned in the yeast expression plasmid pYX212, transformed into the *S. cerevisiae* mutant strain CC362-2A (*leu2*, *ura3*, *met16*; kindly provided by Yolande Surdin-Kerjan, Laboratoire d’enzymologie du CNRS Gif-sur-Yvette, France)^45^ and tested for its ability to suppress the methionine auxotrophy phenotype of such strain. The homologous *S. cerevisiae* PAPS reductase gene (*MET16*) was also cloned in the pYX212 vector and used as a positive control. Functional complementation ability was assessed by serial dilution spot assays carried out in SD medium with and without methionine supplementation. To this end, freshly grown yeast colonies were suspended in sterile double-distilled water and diluted to a starting concentration corresponding to an OD_600_ of 1.0. Four 10-fold dilutions were set-up and tested for each culture/transformant condition, including a negative control strain transformed with the empty pYX212vector, and 2 μl of each cell dilution were spotted on agar plates that were then incubated at 30 °C for 2–3 days.

### PAPS-red transcript quantification in sulfur-deprived *T. melanosporum* mycelia

The *T. melanosporum* Mel28 strain was grown in the dark at 23 °C on a synthetic solid medium (SSM) containing (mg l^-1^) CaCl_2_ 2H_2_O (66), NaCl (25), KH_2_PO_4_ (500), (NH_4_)_2_HPO_4_ (250), MgSO_4_ 7H_2_O (150), FeNa–EDTA (20), thiamine hydrochloride (1), plus agar (15 g/L) and glucose (5 g/L), as described before^46^. To ease medium replacement, mycelia were grown on semi-permeable cellophane membranes (Bio-Rad). For sulfur deprivation experiments, mycelia were first cultured on complete SSM (40 days, t_0_) and then transferred for 7 or 14 days to S-deprived liquid SSM (MgSO_4_ 7H_2_O replaced by the corresponding chloride salt) or to complete SSM before harvesting. Following extraction using the RNeasy plant mini kit with on column DNAse I treatment (Qiagen, Hilden, Germany), RNA samples (2 μg each) were denatured for 5 min at 65 °C in the presence of 1 μl of oligo-dT_12–16_ (500 ng/μl) and 1 μl of a 10 mM dNTP mix. For reverse transcription (RT), the reaction volume was brought to 20 μl with the addition of 4 μl of 5X First-Strand Buffer, 2 μl of 0.1 M DTT, 1 μl of RNaseOUT™ and 200 units of SuperScript™ II reverse transcriptase, incubated for 50 minutes at 42 °C, followed by enzyme inactivation by heating for 15 minutes at 70 °C. Real-time RT-PCR reactions, performed with the Power SYBR® Green PCR Master Mix (Applied Biosystems®), were assembled according to the manufacturer’s instructions and contained the oligonucleotide primers (designed on the *Tm*PAPS-red A gene) *Tm*PAPS-Fwd: 5′-CAAGAGCAAAAGAAGAATGGAGA-3′ and *Tm*PAPS-Rev: 5′-AGATGGCCCATTGTAACCCG-3′ for PAPS reductase and *Tm*18SFwd: 5′-ACTGGTCCGGCCGGATCTT-3′and *Tm*18SRev: 5′-TTCAAAGTAAAAGTCCTGGTTCCC-3′ for the 18S RNA internal standard. Real-time RT-PCR was performed with an AB7300 (Applied Biosystems) apparatus under the following conditions: 10 min denaturation at 95 °C, followed by 40 cycles at 95 °C for 30 s, 57 °C for 30 s, and 72 °C for 45 s. The comparative CT method was used to determine the relative amounts of each transcript, with the 18S rRNA as internal standard. Mean 2−ΔΔCT (ΔCT = CT*gene of interest* – CT*internal standard*) values derived from at least three independent replicates were calculated and analyzed for statistical significance by the Student’s t test.

### Transcriptional Activator Trap assay

The TAT assay was conducted as previously described^14^,^15^. Briefly, the sequences of interest were amplified by PCR and cloned in the pDEST™32 vector (Invitrogen Corporation, Carlsbad, California, USA) in frame with the yeast Gal4-DBD using the Gateway® system (Invitrogen™). The resulting constructs were used to transform the yeast strain MaV103 (*MATa, leu2-3,112, trp1-901, his3*Δ*200, ade2-101, gal4*Δ*, gal80*Δ*, SPAL10::URA3, GAL1::lacZ, HIS3UAS GAL1::HIS3@LYS2, can1R, cyh2R*), followed by plating on SD–minus Leu medium. After 3–5 days at 30 °C colonies were isolated and analyzed by serial dilution assays (starting from an OD_600_ of 1.0) in order to determine *HIS3* and *LacZ* (β-Gal) reporter gene activation ability. For the *HIS3* gene reporter, 2 μl of each dilution were plated on selective plates lacking both leucine and histidine and supplemented with increasing concentrations of the His3 enzyme inhibitor 3-amino-triazole (3-AT); histidine-containing, SD-minus Leu plates were used as positive growth controls. Vectors provided in the ProQuest™ Two Hybrid System kit (Invitrogen™) were used to set up the necessary internal assay controls; wt: strong interaction; m1: weak interaction; and m2: no interaction. Yeast cells transformed with the empty pDEST™32 vector were also included as negative controls. Plates were incubated at 30 °C and yeast growth was checked after two-four days. For the *LacZ* (β-Gal) gene reporter assay, yeast cell dilutions (OD_600_ = 0.1) were spotted on YPD plates overlaid by a nylon membrane, which were then incubated overnight at 30 °C with the cell-loaded surface upward, prior to β-galactosidase assay^47^.

### Nuclear Transportation Trap (NTT) Assay

TAT-positive clones were subcloned into the pNIA-CEN-MBP vector^25^ in frame with the chimeric transcription factor LexA-DBD/yGal4-AD and the resulting constructs were used to transform the L40 yeast strain (*MAT a, trp1, leu2, his3, LYS2::lex A-HIS3, URA3::lex A-lacZ*). Following transformant selection on SD-minus Leu medium, expression of the *HIS3* and *LacZ*/β-Gal reporter genes was analyzed as described for the TAT assay.

### Fluorescence microscopy analysis

The coding regions of the PAPS-reductase genes of *T. melanosporum*, *N. crassa* and *S. cerevisiae* were fused in frame to the C-terminal end of eGFP in the pYX212 vector. The resulting constructs were used to transform the mitochondrial DNA-depleted, rho^0^ yeast strain W303A (*MATa, leu2-3,112 trp1-1 can1-100 ura3-1 ade2-1 his3-11,15*), followed by transformant selection on SD-minus Ura medium. GFP-PAPS reductase transformants were then grown to mid log phase (OD_600_ ~ 0.5) in SD-minus Ura liquid medium. Prior to microscopy analysis, DAPI (1 mg/ml stock solution) was added to 400 μl cell suspension aliquots to a final concentration of 2.5 μg/ml. After incubation at 30 °C for 30 minutes, cells were sedimented by centrifugation for 2 min at 8000 rpm, washed twice in PBS, resuspended in 200 μl of phosphate-buffered saline (PBS) and visualized with a Zeiss Observer 2.1 fluorescence microscope (630X magnification) using the AxioVision Rel 4.8 software.

### Site-directed mutagenesis

The substitution of the three hydrophobic amino acid residues of the PAPS-red A C-terminal polypeptide with the hydrophilic residues present at equivalent positions of the corresponding region of PAPS-red B was performed by PCR using the following oligonucleotides as mutagenic primers: FW: 5′-**GGGGACAAGTTTGTACAAAAAAGCAGGCTTA**ATGACAGTAACTCCAACTGA, RE: 5′-**GGGACCACTTTGTACAAGAAAGCTGGGTA**CTAGATGTCCCGTTGTAACTCGACTTCGTG-3′, where the Gateway cassette sequences are in bold and substituted nucleotides are underlined. The resulting amplicon was first cloned into pDONR222 and subsequently into pDEST32, using the Gateway® system (Invitrogen™).

### Sequence analysis, secondary structure prediction and RNA-seq data analysis

Sequence alignments were performed with MEGA5^48^ and visualized using GeneDoc^49^. The mispredicted GSTUMT00002663001 gene model (PAPS-red A) was corrected by aligning with the cDNA sequencing derived from the TAT screen^14^. RNA-seq data analysis, using the corrected PAPS-red A coordinates, was performed as described^50^.

The Quick2D program (Bioinformatics Toolkit Max Planck Institute http://toolkit.tuebingen.mpg.de/) was used to predict secondary structure on PAPS-red A amino acid sequence, using default settings. Helical wheel projection were drawn at http://kael.net/helical.htm.

### DNA binding assays

PAPS-red A for electrophoretic mobility shift assays (EMSA) and protein binding microarray (PBM) experiments was expressed in auto-induced^51^ *E.coli* SoluBL21™ cells (Genlantis) as a glutathione-S transferase (GST) fusion, purified by affinity chromatography on a Glutathione Sepharose™ 4B column (GE Healthcare) following the manufacturer’s instructions, and exchanged into 10 mM Tris-HCl pH 8.0 buffer containing 120 mM KCl and 1 mM DTT. A fluorescently labeled DNA probe for EMSA analysis, aimed at determining the ability of PAPS-red A to bind DNA at least non-specifically, was prepared by amplification of a 270 bp sequence from the pDONR™222 plasmid (Invitrogen™). The 5′-GTCGACTACAGGTCACTAATACCA-3′ oligonucleotide and the 5′ Dye-682-modified 5′-TGTAATACGACTCACTATAGGG-3′ oligonucleotide were used as forward and reverse primers, respectively. Increasing amounts (0, 100, 200, 300, and 400 ng) of the GST-PAPS-red A fusion protein and of the control GST protein were mixed with 4 ng of the fluorescent DNA probe, followed by the addition of 2 μl of 5X EMSA buffer (100 mM HEPES pH 7.5, 500 mM KCl, 10 mM MgCl_2_, 25% glycerol, 0.5 mM EDTA) into a final volume of 10 μl. Reaction mixtures were then incubated for 30 min at room temperature and electrophoresed on 8% non-denaturing polyacrylamide gels (0.5X TBE buffer; 75 V, 90 min at 4 °C), followed by visualization with an Odyssey CLx Infrared Imaging System (LI-COR Biosciences).

Different concentrations (100, 270 and 700 nM) of the same recombinant GST-PAPS-red A fusion protein were used for PBM experiments. To this end, individual protein samples were applied to two types of arrays harboring different double-stranded oligonucleotide probe sets, designated as “ME” and “HK”, followed by GST-PAPS-red A detection with an anti-GST antibody^52^, (but see also Berger and Bulyk^53^ for a more detailed description of the PBM assay).

## Additional Information

**How to cite this article**: Levati, E. *et al.* Moonlighting transcriptional activation function of a fungal sulfur metabolism enzyme. *Sci. Rep.* **6**, 25165; doi: 10.1038/srep25165 (2016).

## Supplementary Material

Supplementary Information

## Figures and Tables

**Table 1:** Additional TAT and NTT assay-positive, candidate moonlighting proteins from *T. melanosporum*.

Gene model^a^	Protein name	Description^b^	Organism^b^	e-value^b^
GSTUMT00003049001	Mtd1	5,10-methylenetetrahydrafolate dehydrogenase	*Saccharomyces cerevisiae*	3E-99
GSTUMT00005390001	Maf1	mitogen-activated protein kinase MAF1	*Aspergillus flavus*	8E-63
GSTUMT00008696001	Zwf1	glucose-6-phosphate 1-dehydrogenase	*Talaromyces stipitatus*	0
GSTUMT00001950001	Ltv1	low-temperature viability protein ltv1	*Penicillium marneffei*	9E-73
GSTUMT00002382001	Pex20	peroxin 20	*Ajellomyces dermatitidis*	5E-25
GSTUMT00007946001	—	—	—	—

^a^TAT-positive (*LacZ* and *HIS3* reporter gene activation) *T. melanosporum* proteins capable of autonomous nuclear entry; all proteins restored histidine prototrophy and conferred resistance to 50 mM 3-AT concentration.

^b^BLASTP results for each candidate moonlighting protein.
