# Supplementary material for: Moonlighting transcriptional activation function of a fungal sulfur metabolism enzyme
Source: Sci Rep. 2016 Apr 28;6:25165. doi: 10.1038/srep25165 (PMC4848566; doi:10.1038/srep25165)
Supplement: Supplementary Information [file srep25165-s1.pdf]

Moonlighting transcriptional activation function of a fungal sulfur metabolism enzyme

Elisabetta Levati[1], Sara Sartini[1], Angelo Bolchi[1], Simone Ottonello[1,*] and Barbara Montanini[1,*]

[1] Biochemistry and Molecular Biology Unit, Laboratory of Functional Genomics and Protein Engineering, Department of Life Sciences, University of Parma, Parco Area delle Scienze 23/A, 43124 Parma, Italy.

[*] simone.ottonello@unipr.it

[*] barbara.montanini@unipr.it

[*] these authors contributed equally as senior authors

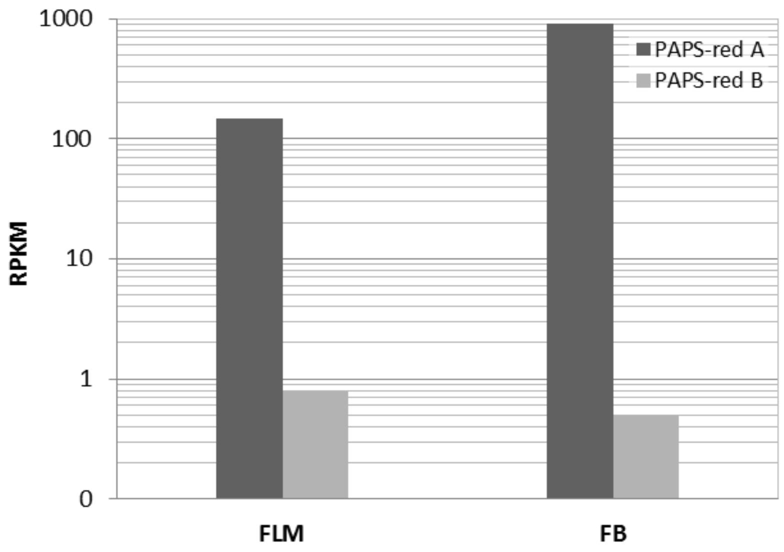

Supplementary Figure S1. Expression levels of *T. melanosporum* PAPS reductases (PAPS-red A and B) in different life cycle stages. RPKM: Reads Per Kilobase per Million mapped reads, plotted on a logarithmic scale. FLM: free-living mycelium; FB: fruiting-body

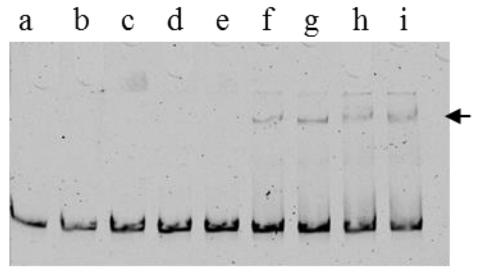

Supplementary Figure S2. Gel mobility-shift assay of PAPS-red A in presence of DNA. Purified, recombinantly expressed GST-PAPS-red A fusion protein (100, 200, 300 and 400 ng; *lanes f-i*, respectively) was tested for its ability to bind DNA by incubation with a fixed amount (4 ng) of a fluorescently labeled *270bp* DNA fragment. GST alone (same amounts as GST-PAPS-red A; *lanes b-e*) and DNA without any added protein served as controls for this experiment. A gel-shifted DNA band only detected in *lanes f-i* and corresponding to a more slowly migrating GST-PAPS-red A-DNA complex, is marked with an arrow.
